# Developmental plasticity increases at the northern range margin in a warm‐dependent amphibian

**DOI:** 10.1111/eva.12349

**Published:** 2016-01-25

**Authors:** Germán Orizaola, Anssi Laurila

**Affiliations:** ^1^ Animal EcologyDepartment of Ecology and GeneticsEvolutionary Biology CentreUppsala UniversityUppsalaSweden

**Keywords:** climate change, ecological modelling, intraspecific variation, species distribution, temperature

## Abstract

Accurate predictions regarding how climate change affects species and populations are crucial for the development of effective conservation measures. However, models forecasting the impact of climate change on natural environments do not often consider the geographic variation of an organism's life history. We examined variation in developmental plasticity to changing temperature in the pool frog (*Pelophylax lessonae*) across its distribution by studying populations from central areas (Poland), edge populations (Latvia) and northern marginal populations (Sweden). Relative to central and edge populations, northern populations experience lower and less variable temperature and fewer episodes of warm weather during larval development. Plasticity in larval life‐history traits was highest at the northern range margin: larvae from marginal populations shortened larval period and increased growth rate more than larvae from central and edge populations when reared at high temperature. Maintaining high growth and development under the scarce spells of warm weather is likely adaptive for high‐latitude populations. The detection of high levels of developmental plasticity in isolated, marginal populations suggests that they may be better able to respond to the temperature regimes expected under climate change than often predicted, reflecting the need to incorporate geographic variation in life‐history traits into models forecasting responses to environmental change.

## Introduction

Climate change is altering entire ecosystems by affecting species distributions, life histories and community dynamics (Parmesan 2006). Thus, there is an urgent need to understand the mechanisms that influence the persistence of populations and species in the face of the ecological changes predicted under climate change. To persist under changing environmental conditions, organisms can respond through migration, plasticity and/or genetic adaptation. If local environmental conditions change, some organisms can disperse to new areas in order to track their optimal environment. However, due to habitat fragmentation and low dispersal capacities, many species must respond to environmental change *in situ* (Chevin et al. 2010). This requires either changes in the genetic constitution of the population as a consequence of adaptive evolution, or plastic responses that adjust the phenotype to the new environment without changes in genetic composition of the population, at least in the short term (Merilä and Hendry 2014). Recent studies have suggested that many observed adaptations to climate change actually result from plastic responses rather than genetic adaptation (Gienapp et al. 2008; Merilä and Hendry 2014). Plasticity, in particular, may play a key role in the initial steps of the adaptation to rapid environmental change when genetic adaptation, a typically slower process that may span many generations, is unable to generate optimal phenotypes at the required pace (Gomez‐Mestre and Jovani 2013).

Forecasting the impact of climate change on natural environments is crucial for setting conservation and management priorities. Recent responses to contemporary climate change include poleward and upward shifts in the distribution of many species (Lenoir and Svenning 2015). Many studies have attempted to predict how the distribution of species and populations can be affected by the expected rate of environmental change (e.g. Guisan and Thuiller 2005; Heikkinen et al. 2006; Elith and Leathwick 2009; Thuiller et al. 2011). Most of these predictions are based on bioclimatic envelope models that use information on different climatic variables and the current distribution of the studied species or populations (Heikkinen et al. 2006). However, in order to make accurate predictions when modelling population responses to climate change, we need to carefully account for dynamic eco‐evolutionary parameters such as the potential of natural populations to respond plastically, or via genetic adaptation (Chevin et al. 2010). Regarding plasticity, plastic responses vary across environmental gradients, and when environmental cues provide a reliable indicator of appropriate adaptive phenotypes, higher plasticity is expected in populations exposed to higher environmental variability (Pigliucci 2001; Hendry 2016). However, only a few studies have assessed how plasticity differs between different areas of a species' distribution (reviewed in Valladares et al. 2014). Five different scenarios of intraspecific variation in phenotypic plasticity can be identified: (i) equal plasticity and no local adaptation across the species distribution, (ii) local adaptation across the range but equal plasticity, (iii) higher plasticity in central areas, (iv) higher plasticity at leading edges and (v) higher plasticity at margins (Valladares et al. 2014). Understanding the patterns of intraspecific variation in plasticity, and including this variation into ecological modelling, is thus essential for accurately predicting the chances of a population to persist when facing rapid climate‐induced environmental change.

Populations occurring at the margins of a distribution are crucial for determining how a species might respond to climate change. These populations are the ones more likely to disappear or to expand as a consequence of changes in climatic conditions. Marginal populations, located at the limits of a species distribution, are often small and might lose quantitative genetic variation more rapidly due to increased genetic drift, founder events and population bottlenecks, which might limit their capacity to develop plastic responses (Ellstrand and Elam 1993; Smith et al. 2006; Willi et al. 2006; but see, e. g. Wood et al. 2015). However, these populations may also harbour unique genetic diversity due to local adaptation processes (Hampe and Petit 2005; Rehm et al. 2015). Furthermore, marginal populations are frequently exposed to higher environmental variability than central ones, which should favour the maintenance of higher levels of phenotypic plasticity (Chevin and Lande 2011). Importantly, marginal populations are often threatened and the subject of major conservation efforts; understanding their capacity to face environmental change through, for example, phenotypic plasticity is crucial for the design of effective conservation plans.

Amphibians are good study models to examine the variation of phenotypic plasticity across species distributions. Many amphibians have wide distributions and high levels of developmental plasticity, especially at the larval stage (Urban et al. 2014). They are also highly suitable for assessing plastic responses across different populations as their size, aquatic habitats and feeding requirements during the larval stage facilitate their rearing under controlled, common garden, conditions. Amphibians are the most highly endangered vertebrate group, with more than 40% of the known species in serious decline (Stuart et al. 2004), so investigating their ability to cope with future environmental change such as climate change is especially timely.

Here, we performed a common garden experiment using populations of the pool frog (*Pelophylax lessonae* Camerano) collected across different areas of the distribution in Europe. We tested for differences in temperature‐induced plasticity in crucial larval development traits between central, edge and marginal populations. Previous studies reported that populations at the northern marginal area of *P. lessonae* distribution showed low levels of genetic diversity (Sjögren 1991; Zeisset and Beebee 2001) and high levels of microgeographic variation in temperature‐induced plasticity (Orizaola and Laurila 2009). The Swedish marginal population of *P. lessonae* is also exposed to lower early spring temperatures than central and edge ones, resulting in a shorter growth season for the larvae (Orizaola et al. 2010). Therefore, we predict that populations living at the northern range margin will maintain a higher degree of temperature‐induced plasticity than populations in central or edge areas to cope with more stringent time constraints during the larval stage. Alternatively, northern populations, experiencing shorter growing seasons, may have evolved overall faster developmental rates rather than greater plasticity.

## Material and methods

**Figure 1:**
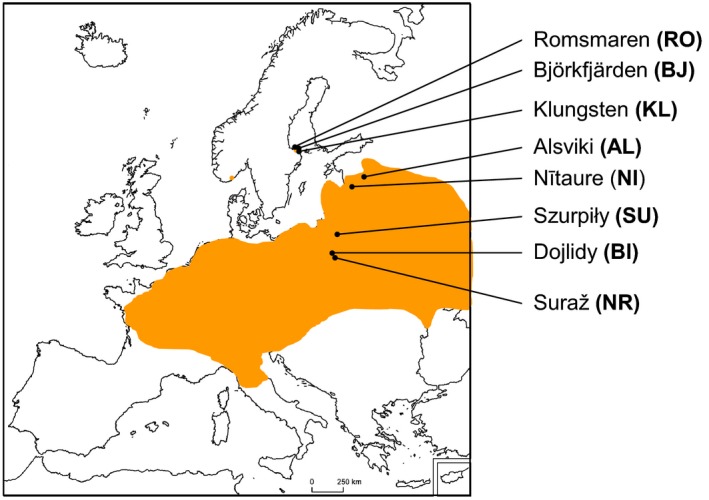
Distribution of *Pelophylax lessonae* in Europe (shaded area) including the populations used in the study.

The pool frog (*P. lessonae*) is abundant and widespread across Europe (Fig. 1), being common in much of Central Europe while maintaining small, relict and isolated populations in Scandinavia (Zeisset and Beebee 2001). *P. lessonae* is a highly warmth‐dependent species, requiring water temperatures over 16°C to initiate breeding activity (Orizaola and Laurila 2009). Breeding occurs in late spring and the larval period lasts ca. 2–3 months. The species also suffers from episodes of total breeding failure in high‐latitude areas during years with poor climatic conditions (i.e. low temperature; Sjögren 1988).

We selected eight *P. lessonae* populations for the study (Fig. 1; Table 1): three populations from Poland (centre of the distribution), two from Latvia (edge populations) and three from Sweden (marginal northern populations). The Swedish population is a relic of postglacial migrations, becoming isolated from the main distribution area during the cooling period around 10.000 BP (Zeisset and Beebee 2001). The present Swedish population is isolated by more than 300 km from the closest continental populations located in Estonia (Orizaola et al. 2010; Fig. 1). Edge populations in Latvia are at the limit of the main distribution area of the species, but still a part of the continuous range (Fig. 1). All the populations were located in medium‐size (<1 ha) permanent water bodies holding a diverse and abundant community of predators, representing the typical breeding habitat for the species. Southern edge or marginal populations were not considered in the study as temperature is not a limiting factor for larval growth and development for these populations and, thus, putative differences in temperature‐induced plasticity are not comparable. In southern populations, pond desiccation, predation pressure, or intra‐ and interspecific competition during the larval stage are likely the most important selective factors acting on these populations.

**Table 1:** Descriptive information of the eight pool frog (*Pelophylax lessonae*) populations included in the study

Population	Code	Region	Area	Geographic coordinates	Date of egg collection
Suraż	NR	Narwianski	Poland	52°58′N 22°58′E	26 May 2006
Szurpiły	SU	Suwalski	Poland	54°14′N 22°53′E	27 May 2006
Dojlidy	BI	Białystok	Poland	53°06′N 23°12′E	27 May 2006
Nītaure	NI	Central Latvia	Latvia	57°04′N 25°11′E	30 May 2006
Alsviki	AL	East Latvia	Latvia	57°27′N 26°56′E	31 May 2006
Björkfjärden	BJ	Uppland	Sweden	60°29′N 18°00′E	6 June 2006
Klungsten	KL	Uppland	Sweden	60°32′N 18°01′E	6 June 2006
Romsmaren	RO	Uppland	Sweden	60°35′N 17°56′E	7 June 2006

We collected freshly laid eggs at the onset of breeding in the eight populations (Table 1). Eggs from ten clumps per population were brought to the laboratory in Uppsala, where they were maintained at room temperature (22°C) until hatching. Each clump was considered a unique family as only a very small fraction of males (ca. 5%) achieves more than one mating. Furthermore, eggs were always collected from clumps separated by at least 5 m to reduce the risk of multiply sampling eggs laid by the same female as pool frog females may lay several clumps close to each other during a breeding season (Sjögren 1988). Due to mortality during the embryonic development period, only seven families were used for BI, and nine for NR and NI populations. When larvae had completed absorption of the gills (Gosner developmental stage 25; Gosner 1960), sixteen larvae from each family were haphazardly selected and placed individually into 1‐L plastic vials, with eight larvae allotted to each of two temperature treatments: 19 and 26°C. These temperatures represent the extremes of the normal range of temperatures experienced by the Swedish populations during larval development (Orizaola and Laurila 2009). The experiment was conducted using water bath systems, which were filled to a depth of 6 cm; heating or cooling units were used to keep the temperature at the desired level (19°C, 19.52 ± 0.07°C and, 26°C; 26.62 ± 0.02°C). Reconstituted soft water (see Orizaola and Laurila 2009 for details) was used during the experiment to assure homogeneous water quality. Lighting was set at 18:6 h light/dark rhythm, corresponding to late spring–early summer conditions in the study area. Tadpoles were fed *ad libitum* with chopped and lightly boiled spinach. Water in the vials was changed completely, and the food renewed every third day.

When the tadpoles approached metamorphosis (emergence of forelimbs, Gosner stage 42), the vials were checked daily and metamorphs were removed. The duration of the larval period was estimated as the number of days elapsed between the start of the experiment and metamorphosis. Mass at metamorphosis was measured to the nearest 0.1 mg with a digital balance after gently blotting the metamorphs in a paper towel to remove excess water. Average daily growth rate was calculated as the mass at metamorphosis divided by the duration of the larval period. Trait plasticity was calculated as the difference in family mean values between the two temperature treatments. This is a robust method for examining plastic responses (Valladares et al. 2006), and in the present data set, it is highly correlated with other plasticity estimates (e.g. correlation of the absolute plasticity estimate with the relative plasticity index: Pearson's *r* > 0.92 in all cases).

We also examined temperature characteristics of the three areas during the months in which *P. lessonae* larvae develop (June–August), using information compiled by www.tutiempo.net. We selected three weather stations situated in the vicinity of the study ponds: Białystok for Poland (53°06′N 23°12′E), Riga for Latvia (56°96′N 24°05′E) and Örskär for Sweden (60°53′N 18°38′E). Specifically, we considered daily average temperature, within‐ and among‐year variation in temperature, and the number of days with high temperature (maximum temperature >25°C) during June–August for the period 1997–2006. Among‐year temperature variation was scored as the median absolute deviation (MAD) of the mean yearly temperature relative to the grand mean for all years (see Nilsson‐Örtman et al. 2012 for a similar approach), and within‐year temperature variation as the difference in daily temperatures (yearly difference between the highest and lowest daily temperature calculated for all years).

Differences in plasticity were examined using mixed‐model anovas where geographic area was included as a fixed factor in the model and population nested within area as a random factor. Models were fitted using restricted maximum‐likelihood (REML) estimations and type III sum of squares. General linear models (GLM) followed by Tukey's HSD post hoc tests were used to examine differences between areas. Differences among geographic areas in average temperature, within‐ and among‐year temperature variation, and days with maximum temperature >25°C were examined on year means with GLMs and Tukey tests. Prior to analysis, normality and homoskedasticity of the data were assessed using Shapiro–Wilk and Levene tests, respectively. All analyses were conducted in SPSS 21.

## Results

**Figure 2:**
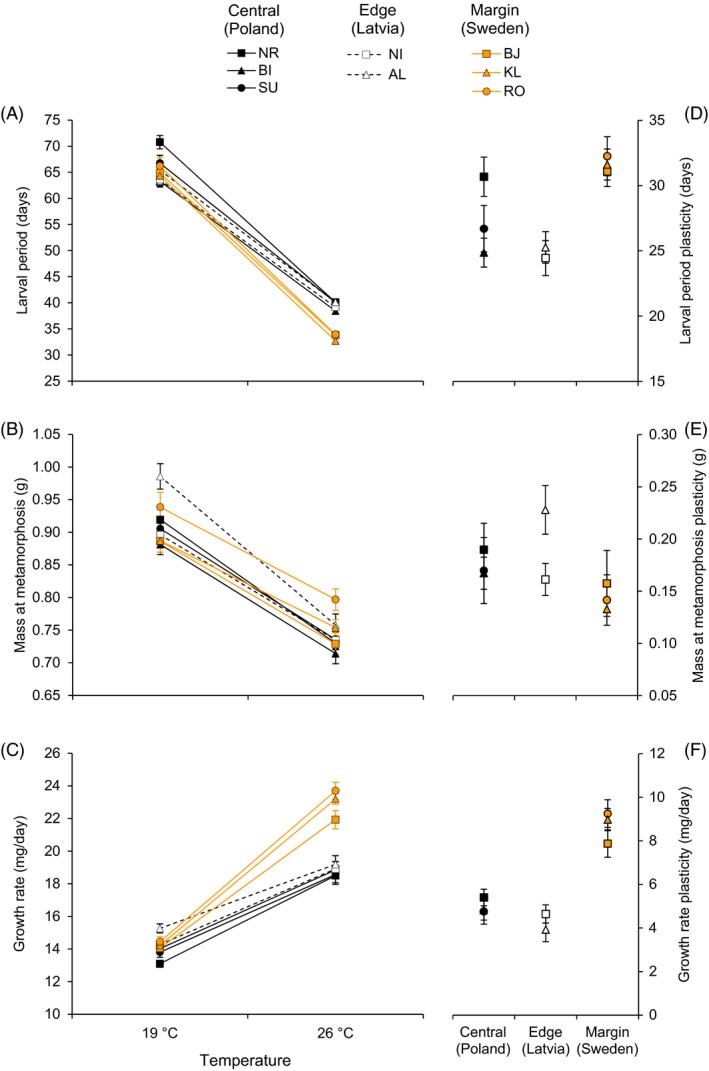
Reaction norms and plasticity for the duration of the larval period (A, D), mass at metamorphosis (B, E) and growth rate (C, F) of *Pelophylax lessonae* from central, edge and northern margin areas reared at two temperatures. See main text for population codes.

Plasticity in the duration of the larval period differed between geographic areas (*F*_3,4.5_ = 540.3, *P* < 0.001), and was higher in populations located at the northern margin of the species distribution (ca. 17% higher on average; Tukey tests, *P* < 0.002; Fig. 2A). Plasticity in mass at metamorphosis also differed between areas (*F*_3,5.1_ = 96.82, *P* < 0.001, Fig. 2B), although posterior GLM and Tukey's tests did not reveal but a nonsignificant difference (*P* = 0.089) between marginal and edge areas. Plasticity in growth rates differed between areas (*F*_3,5.2_ = 297.0, *P* < 0.001), and was much higher in marginal populations (ca. 45% higher on average; Tukey tests, *P* < 0.0001; Fig. 2C). No differences in plasticity were detected between central and edge areas (Tukey tests, *P* > 0.14 in all cases), nor between populations within each geographic area, for any of the studied traits (*P* > 0.4 in all cases).

The geographic areas differed in average temperature (*F*_2,30_ = 6.68, *P* < 0.004), within‐year daily temperature variation (*F*_2,30_ = 141.42, *P* < 0.0001) and the number of days with maximum temperature over 25°C (*F*_2,30_ = 17.88, *P* < 0.0001; *Supplementary information*). Among‐year variation in temperature did not differ among areas (*F*_2,30_ = 0.423, *P* = 0.659). Marginal populations are exposed to overall lower temperatures (15.9°C on average; Tukey tests, *P* < 0.029 in all cases), lower variation in daily temperature (6°C on average; *P* < 0.001 in all cases), and fewer days with high temperature during the larval period (8 days per season on average; *P* < 0.002 in all cases; *Supplementary information*). Central populations experienced the highest variability in daily temperatures (12°C on average; *P* < 0.001 in all cases). Central and edge areas experienced higher average temperatures and also a greater number of days with maximum temperature >25°C (*P* < 0.029 in all cases) than the marginal populations, but there was no significant difference between them (17.4°C and 34 days per season in the central populations and 17.8°C and 25 days per season in the edge populations; *P* = 0.745 and 0.136, respectively; see *Supplementary information*).

## Discussion

We found that developmental plasticity was not uniform across the geographic range of *P. lessonae*. Plasticity was highest in marginal populations, and lowest for the central and edge populations. These results agree with theoretical predictions of more canalized phenotypes in populations located close to the centre of a species distribution, with higher plasticity among marginal populations (Chevin and Lande 2011). Previous studies have shown that organisms living at the edge of the distribution of a species may maintain high plasticity (e.g. Nilsson‐Örtman et al. 2012; Therry et al. 2014; Valladares et al. 2014 and references therein; Lázaro‐Nogal et al. 2015). In amphibians, studies looking at the variation in plastic responses across the distribution of a species have reported mixed results, with either no differences in plasticity or even lower plasticity in high‐latitude populations (e.g. Merilä et al. 2000, 2004). To our knowledge, this is the first study showing higher levels of developmental plasticity in isolated, marginal populations of a vertebrate species.

In our study, differences in plasticity between the marginal and central‐edge areas were a consequence of individuals from the marginal populations shortening larval period and increasing growth rates to a greater extent than individuals from the central and edge areas when reared under more benign conditions (i.e. high temperature). No differences between areas were apparent at the low temperature regime, rejecting the hypothesis of northern populations showing overall faster developmental rates rather than greater plasticity. In amphibians, shorter larval period is usually linked to higher fitness (Semlitsch et al. 1988; Altwegg and Reyer 2003) and is expected to be highly beneficial for time‐constrained species such as those living at high latitudes or under high desiccation risk (Richter‐Boix et al. 2011; Orizaola et al. 2013). Although our experimental design cannot exclude differences among geographic areas in maternal or early environment effects, conditions prior to the start of the experiment were similar among populations and maternal effects appear to be small compared to genetic effects across latitude in other amphibian species (e.g. *R. temporaria*, Laugen et al. 2002).

Theory predicts that higher plasticity should evolve in more heterogeneous environments (Berrigan and Scheiner 2004). However, it is not always easy to identify the key environmental factors that affect the development and maintenance of plastic responses. Our analyses on temperature differences between the three study areas reveal that the marginal populations experienced the lowest average temperature, but also the lowest daily temperature variation. Temperature variation, on the contrary, was highest in central and edge populations. These results agree with previous studies in damselflies that also detected lower within‐year temperature variation towards higher latitudes (Nilsson‐Örtman et al. 2012), and suggest that the overall variation in temperature may not be a factor affecting the maintenance of higher developmental plasticity in northern marginal populations. Interestingly, the northern marginal area differs from central and edge areas for having much fewer episodes of warm weather (maximum temperature >25°C) during the larval growth season. In warmth‐dependent species, such as *P. lessonae*, the capacity to maintain high growth and development rates under favourable conditions (i.e. periods of warm weather) may be crucial for the survival of populations living at northern range limits that are exposed to more challenging climatic conditions. High levels of developmental plasticity in northern marginal populations may, thus, be maintained to allow larvae to maximize growth and development during the sporadic episodes of warm weather in order to metamorphose before environmental conditions deteriorate later in the season.

Fast growth in response to strong time constraints is adaptive and common in nature (e.g. Altwegg 2002; De Block and Stoks 2004; Stoks et al. 2006), although it can be costly for individuals (Dmitriew 2011). Costs can be immediate, for example an increase in predation risk linked to higher activity (Mangel and Munch 2005), or appear later in life, for example increased oxidative stress, reduced immune function, or reduced reproductive output (e.g. De Block and Stoks 2008; Auer et al. 2010). Thus, considering the costs, it is reasonable to expect that only northern marginal populations, which are exposed to less frequent episodes of warm weather and experience overall greater time constraints than central and edge populations, will maintain plastic responses allowing maximal growth and development rates under the warmer environmental conditions.

The lack of genetic variation typical of many populations living in marginal areas has been pointed out as a classic constraint for the evolution and maintenance of high levels of plasticity (Schlichting and Pigliucci 1998; Murren et al. 2015). Interestingly, we detected the highest levels of developmental plasticity in the marginal Swedish pool frog populations, which harbour very low variability in genetic markers (Sjögren 1991; Zeisset and Beebee 2001). These results reinforce the view that the lack of neutral genetic variation may not be a good proxy for estimating the degree of developmental plasticity for a population (Reed and Frankham 2001). Recent studies have also revealed that some small populations may harbour similar levels of adaptive genetic variation and plasticity as larger ones (Wood and Fraser 2014; Wood et al. 2015), suggesting that the use of the variation in neutral markers as an indicator of the capacity of a population to respond to environmental change is not straightforward.

Developmental plasticity is widespread in nature and has a clear role in facilitating adaptation to climate change (Merilä and Hendry 2014; see Urban et al. 2014 for amphibians). However, only a handful of studies have evaluated intraspecific variation in developmental plasticity across broad geographic scales, and none has yet considered isolated, marginal populations (e.g. Valladares et al. 2014; and references therein). Understanding the variation of plastic responses across the distributions of species is crucial for the correct assessment of the impact that environmental change may have on natural systems. So far, most ecological models attempting to forecast the impact of climate change have adopted a static approach regarding plasticity, considering that all populations of a species exhibit the same levels of plastic responses (Valladares et al. 2014; but see Chevin et al. 2010).

We acknowledge that the maintenance of high levels of developmental plasticity under naturally occurring conditions does not directly translate into an advantage of plasticity under climate change conditions. However, as adaptive plasticity can contribute to the preservation of genetic variation within a population, plasticity should be viewed as an adaptive process that can provide substrate for rapid adaptation to novel environmental conditions (Gomez‐Mestre and Jovani 2013).

Our study indicates that not accounting for variation in plasticity within a species can lead to inaccurate predictions about the vulnerability of populations to environmental change. In particular, our results suggest that populations at the geographic margins of the distribution may be more capable of buffering environmental change than previously expected, which may have important implications for modelling biotic responses to environmental change and for the design of conservation measures. For example, the capacity of small and isolated populations to maintain plastic responses and adapt *in situ* to environmental change may reinforce the value of nature reserves and other protected areas as an effective measure to preserve biodiversity in marginal habitats (see, e.g. Gillingham et al. 2015). However, we call for more studies on the geographic variation of plastic responses to clarify how general this trend is in nature, as well as to what extent it allows organisms to cope with the current pace of environmental change.

## Data archiving

Life‐history and temperature data for this study are available at ResearchGate at https://www.researchgate.net/publication/284420870_OrizaolaLaurila_EvolApp2016_DATA

## Supporting information


**Figure S1.** Temperature characteristics of the study areas (see main text for details).
**Table S1.** Mixed models ANOVAS and univariate general linear models for life‐history traits.
**Table S2.** Univariate general linear models for temperature variables.
