# Supplementary material for: Developmental plasticity increases at the northern range margin in a warm‐dependent amphibian
Source: Evol Appl. 2016 Jan 25;9(3):471–8. doi: 10.1111/eva.12349 (PMC4778106; doi:10.1111/eva.12349)
Supplement: Supplementary file 1 — Figure S1. Temperature characteristics of the study areas (see main text for details). Table S1. Mixed models ANOVAS and univariate general linear models for life‐history traits. Table S2. Univariate general linear models for temperature variables. [file EVA-9-471-s001.docx]

**Developmental plasticity increases at the northern range margin in a warm-dependent amphibian**

**Supplementary information.**

**Fig. S1.** Temperature characteristics of the study areas (see main text for details).

**(A)** Daily average temperature June-August 1997-2006

**(B)** Within-year daily temperature variation June-August 1997-2006

**(C)** Among-year temperature variation June-August 1997-2006

**(D)** Days with maximum temperature > 25 °C June-August 1997-2006

**Table S1.** Mixed models ANOVAS and univariate general linear models for life-history traits

**(A)** Larval period

|  | dfN | dfD | *F* | *P* |  |
| --- | --- | --- | --- | --- | --- |
| Area | 3 | 4.493 | 540.349 | < 0.001 |  |
|  |  |  |  |  |  |
|  | Variance ± SE | | *Z* | *P* |  |
| Population (Area) | 2.078 ± 2.697 | | 0.770 | 0.441 |  |
|  |  |  |  |  |  |
|  |  |  |  |  |  |
| Univariate GLM | |  |  |  |  |
|  |  |  |  |  |  |
|  | SS | df | MS | *F* | *P* |
| Area | 0.028 | 2 | 0.014 | 2.498 | 0.089 |
| Error | 0.403 | 72 | 0.006 |  |  |
| Total | 2.544 | 75 |  |  |  |
|  |  |  |  |  |  |
|  |  |  |  |  |  |
| **(B)** Mass at metamorphosis | | |  |  |  |
|  | dfN | dfD | *F* | *P* |  |
| Area | 3 | 5.018 | 96.821 | < 0.001 |  |
|  |  |  |  |  |  |
|  | Variance ± SE | | *Z* | *P* |  |
| Population (Area) | 0.0002 ± 0.0005 | | 0.402 | 0.688 |  |
|  |  |  |  |  |  |
|  |  |  |  |  |  |
| Univariate GLM | |  |  |  |  |
|  |  |  |  |  |  |
|  | SS | df | MS | *F* | *P* |
| Area | 0.028 | 2 | 0.014 | 2.498 | 0.089 |
| Error | 0.403 | 72 | 0.006 |  |  |
| Total | 2.544 | 75 |  |  |  |
|  |  |  |  |  |  |
|  |  |  |  |  |  |
| **(C)** Growth rate |  |  |  |  |  |
|  |  |  |  |  |  |
|  | dfN | dfD | *F* | *P* |  |
| Area | 3 | 5.184 | 296.960 | < 0.001 |  |
|  |  |  |  |  |  |
|  | Variance ± SE | | *Z* | *P* |  |
| Population (Area) | 0.091 ± 0.243 | | 0.377 | 0.706 |  |
|  |  |  |  |  |  |
|  |  |  |  |  |  |
| Univariate GLM | |  |  |  |  |
|  |  |  |  |  |  |
|  | SS | df | MS | *F* | *P* |
| Area | 278.922 | 2 | 139.461 | 49.454 | < 0.001 |
| Error | 203.042 | 72 | 2.820 |  |  |
| Total | 3451.851 | 75 |  |  |  |

**Table S2.** Univariate general linear models for temperature variables

**(A)** Daily average temperature June-August 1997-2006

|  | SS | df | MS | *F* | *P* |
| --- | --- | --- | --- | --- | --- |
| Area | 19.233 | 2 | 9.616 | 6.676 | 0.004 |
| Error | 38.892 | 27 | 1.440 |  |  |
| Total | 8767.505 | 30 |  |  |  |

**(B)** Within-year daily temperature variation June-August 1997-2006

|  | SS | df | MS | *F* | *P* |
| --- | --- | --- | --- | --- | --- |
| Area | 185.367 | 2 | 92.684 | 141.416 | < 0.001 |
| Error | 17.696 | 27 | 0.655 |  |  |
| Total | 2816.622 | 30 |  |  |  |

**(C)** Among-year temperature variation (MAD) June-August 1997-2006

|  | SS | df | MS | *F* | *P* |
| --- | --- | --- | --- | --- | --- |
| Area | 0.082 | 2 | 0.041 | 0.423 | 0.659 |
| Error | 2.609 | 27 | 0.097 |  |  |
| Total | 9.739 | 30 |  |  |  |

**(D)** Days with maximum temperature > 25 °C June-August 1997-2006

|  | SS | df | MS | *F* | *P* |
| --- | --- | --- | --- | --- | --- |
| Area | 3445.8 | 2 | 1722.9 | 17.882 | < 0.001 |
| Error | 2601.4 | 27 | 96.348 |  |  |
| Total | 21100 | 30 |  |  |  |
